# Low cross-talk optical addressing of trapped-ion qubits using a novel integrated photonic chip

**DOI:** 10.1038/s41377-024-01542-x

**Published:** 2024-08-20

**Authors:** Ana S. Sotirova, Bangshan Sun, Jamie D. Leppard, Andong Wang, Mohan Wang, Andres Vazquez-Brennan, David P. Nadlinger, Simon Moser, Alexander Jesacher, Chao He, Fabian Pokorny, Martin J. Booth, Christopher J. Ballance

**Affiliations:** 1 University of Oxford, Department of Physics, Oxford, OX1 3PU UK; 2 University of Oxford, Department of Engineering Science, Oxford, OX1 3PJ UK; 3 Institute of Biomedical Physics, Medical University of Innsbruck, Müllerstraße 44, 6020 Innsbruck, Austria

**Keywords:** Physics, Integrated optics, Photonic devices

## Abstract

Individual optical addressing in chains of trapped atomic ions requires the generation of many small, closely spaced beams with low cross-talk. Furthermore, implementing parallel operations necessitates phase, frequency, and amplitude control of each individual beam. Here, we present a scalable method for achieving all of these capabilities using a high-performance integrated photonic chip coupled to a network of optical fibre components. The chip design results in very low cross-talk between neighbouring channels even at the micrometre-scale spacing by implementing a very high refractive index contrast between the channel core and cladding. Furthermore, the photonic chip manufacturing procedure is highly flexible, allowing for the creation of devices with an arbitrary number of channels as well as non-uniform channel spacing at the chip output. We present the system used to integrate the chip within our ion trap apparatus and characterise the performance of the full individual addressing setup using a single trapped ion as a light-field sensor. Our measurements showed intensity cross-talk below ~10^–3^ across the chip, with minimum observed cross-talk as low as ~10^–5^.

## Introduction

Since their original proposal as a platform for quantum information processing^1^, trapped ions have emerged as one of the leading contenders for building a useful quantum computer. To date, small-scale trapped-ion systems have demonstrated the highest single- and two-qubit gate fidelities^2–5^, longest coherence times^6^, and lowest state preparation and measurement errors^7^ of any quantum computing platform. For realising a functional large-scale quantum computer, this level of control needs to be extended to a large number of qubits. This includes the implementation of all necessary quantum operations on the qubit register with very high fidelity, as well as the execution of targeted (addressed) operations on specific subsets of qubits within the register with minimal effect on the unused (idle) qubits in the computation.

Individual addressing in most types of ion trap quantum computing architectures requires locally modifying the interaction between the ions and the radiation used to perform operations with very low cross-talk, where the natural inter-ion spacing is a few micrometres^8,9^. This can be done by altering the properties of the magnetic and/or electric fields experienced by each of the ions to change their response to a globally applied field^10–15^, by focusing down the radiation used to perform quantum gates on each ion^16–22^, or by using a combination of the two approaches. The former approach works with both laser and microwave radiation but requires intricate local control of electric and magnetic fields. Hence, it is only compatible with microfabricated traps with numerous electrodes. The latter approach is only viable when optical wavelengths are used for operations, either when the qubit transition is driven with a two-photon Raman process or the qubit transition itself corresponds to an optical wavelength. It requires focusing down laser beams to micrometre scales, therefore working close to the diffraction limit. However, this method is applicable to all ion trap architectures.

Several methods have been developed for achieving individual optical addressing in ion trap systems, including micro-mirror beam steering^16–18^, acousto-optic deflectors^19^, multi-channel acousto-optic modulators^20^ (AOMs), and micro-lens arrays^21^. These methods vary in cross-talk performance and scalability. Using micro-mirror arrays to steer the lasers onto the target ions offers individual addressing with very low cross-talk. However, the time required to reconfigure the beam positions or to tune the amplitude of the beams is comparable to the timescale of the gate operations, therefore accounting for a significant amount of the sequence run time. Acousto-optic deflectors offer similarly low cross-talk but also enable fast intensity control of the individual beams. However, they lack individual beam frequency control, therefore limiting the set of unitaries that can be implemented in parallel. Furthermore, only a few ions can be addressed at any one time due to power losses in higher diffraction orders. Multi-channel AOMs solve the problem of fast parallel control. However, they exhibit an order of magnitude larger cross-talk compared to the previous two approaches due to electronic cross-talk between channels in existing devices. Additionally, they are only manufactured with a fixed and equal inter-channel spacing, and readily available with only up to 32 channels, limiting both scalability and ability to address non-evenly spaced chains. The micro-lens array approach solves the majority of the problems listed above by enabling parallel operations with very low cross-talk. However, the system presented in Ref. ^21^. has a very high insertion loss limiting the obtainable light intensity at the ion, and a fixed, uniformly spaced output pattern that cannot be easily adapted to non-uniform ion crystals.

In addition to the above approaches, light delivery to trapped ions has also been realised via photonic integrated circuits made by direct laser writing within the trap chip itself^23,24^. However, due to the difference in the mode size between these waveguides and standard optical fibres, interfacing them results in very high coupling losses (typically >10 dB). Furthermore, these devices are typically made out of silicon nitride whose performance is significantly reduced at wavelengths below ~450 nm due to extremely high material absorption, limiting their application to some ion species. Finally, they cannot be easily scaled to individually address many closely-spaced ions in a single chain.

In this work, we demonstrate an approach to individual optical addressing in trapped-ion chains with minimal cross-talk using a network of fibre-coupled modulators connected to a high-performance photonic chip. We employ spherical phase-induced multiscan waveguides^25^ (SPIM-WGs) that provide precise control over the optical mode and enable much higher refractive index (RI) contrast modifications in optical glass compared to conventional ultrafast laser-written waveguides. A similar approach using laser-written waveguide devices has been reported^22^, however matching the output of the waveguides presented in ref.^22^ to an ion chain while maintaining a good spot quality and low cross-talk is yet to be demonstrated.

The photonic chip that we present here adopts individual adiabatic mode converters as light guiding channels that exhibit excellent optical mode confinement and low cross-talk even at a channel separation of a few micrometres. This ensures that the errors due to nearest-neighbour cross-talk do not limit the performance of the trapped-ion device. Furthermore, the manufacturing process of the SPIM-WGs offers high flexibility, facilitating easy modification of the number of channels, the channel positions, and the mode shapes in the chip design, therefore making the chip easily adaptable to the ion configurations found in most ion trap experiments. The use of a fibre network for light delivery to the photonic chip further simplifies the process of exchanging devices by minimising the necessary realignment, thus allowing for rapid iteration of the system design.

## Results

### Photonic chip design

**Fig. 1:**
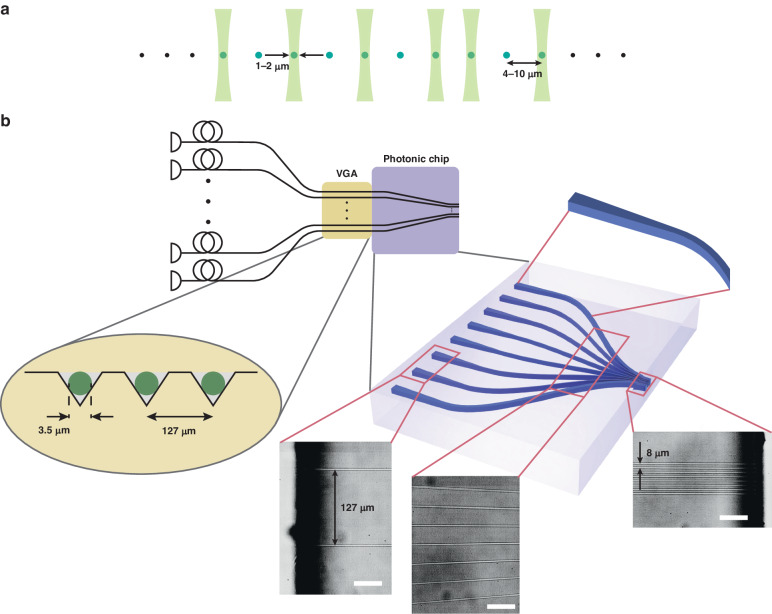
Optical addressing in chains of trapped ions and the diagram of the photonic chip design. **a** Requirements for individual optical addressing in chains of trapped ions. The spacing between the individual laser beams must match the ion separation, typically 4–10 µm^8,9^. The spot size needs to be small enough to minimise the intensity at the neighbouring ions, but larger than the diffraction limit for the laser wavelength in use. **b** A diagram of the photonic chip design. The input light from the V-groove array (VGA), whose output channels are spaced by 127 µm, is coupled into the photonic chip as shown in the top left. Shown in the middle is a 3D representation of a photonic chip featuring eight channels. Each channel serves as a high-efficiency adiabatic mode converter. The optical modes are precisely engineered to satisfy the trapped-ion addressing requirements at the output of the photonic chip, where the channel separation is brought down to 8 µm. The grayscale microscopic images of the channel structures were acquired from the top of the photonic chip. The scale bar is 50 µm. The dark regions at the chip facets were due to reduced microscopic illumination. The device has two straight regions, input (2.2 mm) and output (0.2 mm), which are connected by a curved region (7.2 mm). The adiabatic mode conversion is performed over the 2.2 mm at the channel input and the corresponding region is highlighted at top right of the diagram

Performing laser-driven targeted operations in long chains of trapped ions imposes several competing requirements on the individual addressing setup. First, to minimise errors on the idle qubits, it is crucial to minimise the cross-coupling between neighbouring ion sites. Second, the spacing between neighbouring channels must match the ion-ion spacing, typically on the order of a few micrometres^8,9^. This requires generating a series of closely spaced beams, each focused to an ~1 µm waist radius as shown in Fig. 1a. As a result, this approach necessitates focusing the beams near the diffraction limit. Working with tightly focused beams can also increase errors in the quantum operations due to intensity, phase, and/or polarisation modulation of the light at the ion position. This modulation can be caused by mechanical drifts in the optical system or by the secular motion of the ions^26,27^. Hence, it is desirable to make the beam waist radius as large as possible. The requirements on the beam spacing and beam waist radius constrain the waist-to-spacing ratio of the device output. A larger ratio makes integration into the trapped-ion system easier and more robust to errors but also increases the cross-talk within the device. Furthermore, to ensure that the required input laser power scales favourably with the qubit register size, it is important to maintain a high throughput efficiency of the system. Finally, the ability to control the intensity, phase, and frequency of individual channels in parallel enables the simultaneous application of different unitary operations on different target qubits, therefore reducing the algorithm run time.

To address these challenges, we developed a photonic chip that offers a very high refractive index contrast between the waveguide core and cladding, therefore maintaining a very low level of cross-talk between neighbouring channels even at micrometre scale channel separations. In our system the source laser is coupled into a series of fibre splitters, such that the light is split up into the required number of channels. Each channel is then connected to a fibre AOM that allows for individual phase, frequency, and amplitude control, and for switching of each beam. The fibre AOMs are connected to a fibre V-groove array (VGA) whose output is an array of fibre cores with a mode field diameter (MFD) of 3.5 µm and a uniform spacing of 127 µm. While the output of the VGA provides a suitable spot size, the core spacing is far too large compared to the typical ion-ion spacing. The core spacing could be reduced using a global imaging system with appropriate demagnification. To convert the 127 µm core spacing from the VGA to the required ~4 µm beam spacing at the ion position, the required demagnification would be ≈25. However, this scaling factor would also apply to the beam spot size, resulting in operation below the diffraction limit for the wavelengths typically used in trapped-ion setups (300 nm–2 µm). Hence, the VGA is coupled to the photonic chip as shown in Fig. 1, and fixed in place with glue (details in “Materials and methods”) to avoid misalignment during operation.

The photonic chip performance benefited from several advanced design and fabrication techniques, as detailed in the following sections. It was designed to convert the input optical modes and spacing of the VGA channels into closely spaced (8 µm) modes suitable for trapped-ion addressing. Each channel incorporated a high-efficiency adiabatic mode converter to transform the optical mode from the VGA, equivalent to a single-mode fibre (SMF), into the smaller optical mode required to address the ions. The adiabatic mode conversion was implemented over a distance of 2.2 mm, starting from the chip’s input facet. Subsequently, curved routing of the channels was used to reduce the channel spacing, while maintaining their cross-sectional shape at the output facet of the chip. The microscopic images in Fig. 1 obtained from the top of the photonic chip, capturing the input facet, bending region, and output facet, illustrate the progression of the channel spacing along the chip. Even though the channels were closely stacked with 8 µm spacing over a distance of 0.2 mm, we were able to maintain a low nearest-neighbour cross-talk at a level of ~10^–4^ as demonstrated in the following sections.

### High-contrast refractive index modification

As outlined in the previous section, individual optical addressing of trapped ions requires the generation of closely spaced beams with maximum waist-to-spacing ratio and minimum cross-talk. That way, the errors due to unwanted operations on the idle qubits are minimised, while maximising the robustness of the system. This translates to the need for a very high level of light confinement in the channels of the photonic chip. Existing methods of producing micro-waveguides in photonic chips require a very small waist-to-spacing ratio to satisfy the low cross-talk requirement. An ideal fabrication method would allow an increase in waveguide size while maintaining single-mode operation and low cross-talk. The key to this is a stronger confinement of the mode by increasing the RI contrast between the core and the cladding. This level of RI control has not yet been possible with conventional ultrafast laser writing, where high RI contrast is typically accompanied by poor control of the mode shape.

**Fig. 2:**
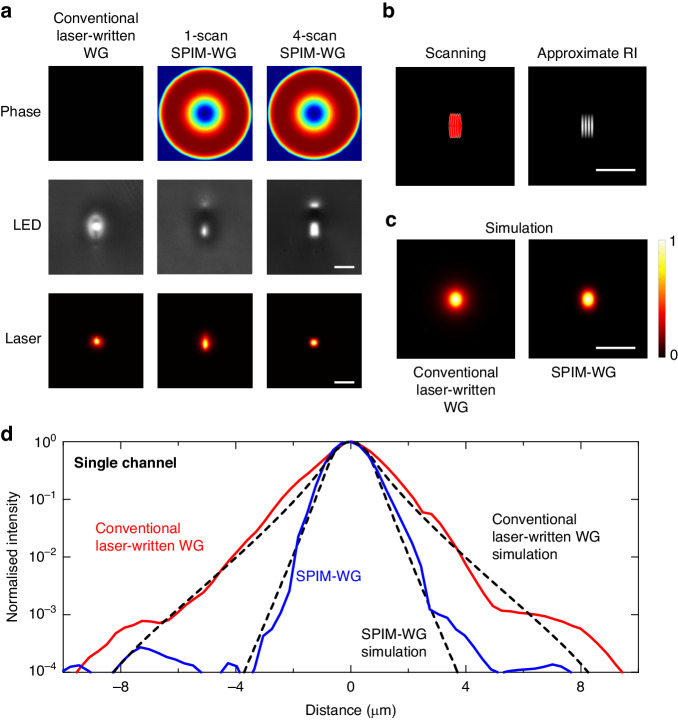
Optical modes at the photonic chip output. The size of the scale bars in (**a**–**c**) is 5 µm. **a** Comparison of waveguide fabrication techniques: single-scan conventional ultrafast laser-written waveguide (left), single-scan SPIM waveguide (middle), and multiscan SPIM waveguide (right). Top: varying optical phases applied to the ultrafast laser system for waveguide inscription. Middle: microscope images of the waveguide facets at the chip output under broadband LED illumination. Bottom: 532 nm laser mode profiles of the waveguides at the chip output (intensity normalized individually). **b** Scanning scheme and approximate RI profile for a single SPIM-WG channel at the chip output. **c** COMSOL-simulated mode profiles for a conventional laser-written waveguide and for the designed SPIM-WG output (intensity normalised individually). **d** HDRMs and COMSOL-simulated optical modes for a conventional laser-written waveguide and for the designed SPIM-WG

In order to address this challenge, in this work we fabricated SPIM-WGs using a multiscan scheme to precisely control the shape and the size of the channel cross-section, enabling single-mode operation and a large core diameter. To achieve high-precision RI modification, the SPIM-WGs were fabricated with combined spherical aberrations of first-order Zernike mode 11 and third-order Zernike mode 37 introduced into the focused laser^28^, with root mean square (RMS) amplitudes of −1 rad and -0.3 rad, respectively. The SPIM-WG design incorporated four scans with a core separation of 0.4 µm forming a single light guiding channel. The RI contrast measured using high-resolution quantitative phase microscopy was ~0.015 (with a core RI of 1.525 and a cladding RI of 1.51), which is two to three times higher than for a waveguide created by conventional ultrafast laser writing^25^. The horizontal core size of one complete SPIM channel was measured to be about 1.8 µm, which was approximately the maximum diameter that maintained single-mode operation with an RI contrast of 0.015 at 532 nm^29^. Figure 2a presents a comparison between LED microscopic images and 532 nm laser mode profiles for a conventional laser-written waveguide, a single-scan SPIM-WG, and a four-scan SPIM-WG. Figure 2b illustrates the scanning scheme and the approximate RI profiles. Additionally, Fig. 2c shows COMSOL-simulated mode profiles for a conventional laser-written waveguide and a four-scan SPIM-WG. We observed a slight mode elongation along the vertical direction. However, this does not affect the mode quality or the cross-talk in the direction parallel to the ion chain. In fact, it is advantageous for optical addressing of trapped ions, as it improves the robustness of the system to intensity modulation in that direction.

We observed a bright lobe above the four-scan SPIM-WG, as shown in Fig. 2a. Such lobes were significantly weaker in the single-scan SPIM-WG. Close examination showed that these lobes possessed a low RI contrast and exhibited high guiding losses. Notably, there was a negative relative RI modification between the main lobe and the upper lobe. In this application, the main lobe was used as the light guiding channel, and we observed no adverse effects arising from the presence of the upper lobe. The strong confinement to the main guiding channel could be attributed to the presence of the region with negative RI contrast between the two lobes, as well as the high transmission loss associated with the upper lobe.

To illustrate the light confinement capabilities, we performed high dynamic range measurements (HDRMs) of the guided laser modes at 532 nm. Figure 2d illustrates measured and simulated HDRMs for a single channel. We observed a close agreement between the simulations and the experiments. A clear difference in the degree of mode confinement was observed between the conventional laser-written waveguide and the SPIM-WG. At distances of 2–8 µm from the centre, the intensity from the SPIM-WG was around an order of magnitude lower compared to that of the conventional laser-written waveguides we tested.

**Fig. 3:**
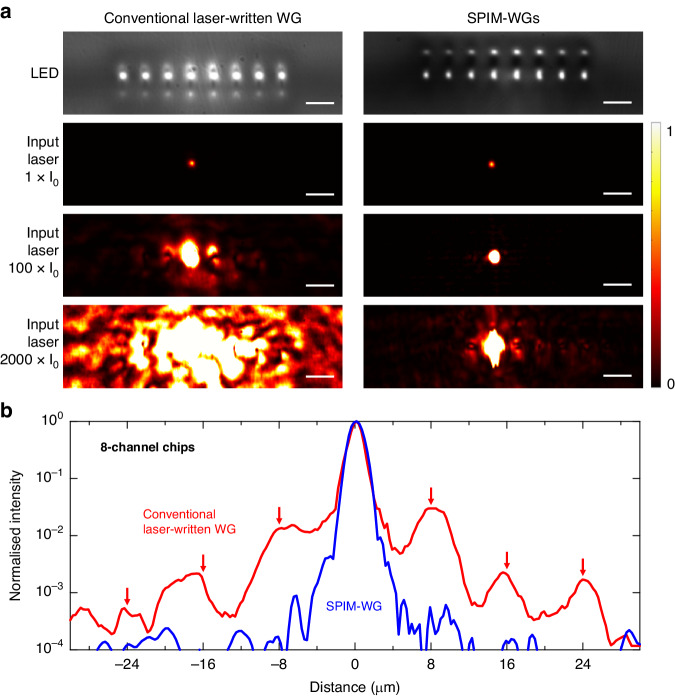
Light confinement properties of the SPIM-WG channels. **a** LED-illuminated microscope images and laser mode profiles for two 8-channel chips fabricated using single-scan conventional laser-written (left) and the SPIM-WG (right) methods. One SMF was coupled to the fourth channel (left to right) of the chip’s input facet. To show effects over a high dynamic range, three input laser intensities were applied (1 × I_0_, 100 × I_0_, and 2000 × I_0_, adjusted using neutral density filters, where I_0_ is the lowest applied input laser intensity). The size of the scale bars is 10 µm. **b** HDRMs for two 8-channel chips, each fabricated using single-scan conventional laser-written or the SPIM-WG methods. The intensity in this measurement is normalised relative to the highest observed intensity. The red arrows mark the positions of the remaining channels

We designed eight-channel chips, with a channel separation of 8 µm, according to the specifications outlined in the previous section and illustrated in Fig. 1. Our SPIM-WG fabrication technique enables a spacing of as low as 2 µm without affecting the mode quality. However, we estimate that at a spacing below 5–6 µm, the cross-talk in the chip would be too high (>10^–3^) and the resulting error would overwhelm the performance of the trapped-ion device. In Fig. 3a we show LED microscopic images of the output chip facet. The outer channels appear dimmer than the central channels due to the lower LED intensity away from the centre. We conducted measurements to assess the overall loss (including coupling and propagation losses) across all waveguide channels, comparing straight waveguides to bent waveguides. We did not see any noticeable difference between the two. This suggests that the bending losses in all channels are negligible, owing to the high RI contrast resulting in a strong mode confinement (see the Supplementary information for further details).

Channel cross-talk was assessed by coupling a 532 nm laser through a single-mode fibre to one central channel at the chip input facet, then observing the full 8-channel intensity distribution at the chip output. High dynamic range measurements were compiled from a sequence of images taken with different calibrated neutral density filters. As shown in Fig. 3a, the SPIM-WGs showed far better confinement of the laser light to the vicinity of the channel compared to conventional laser-written waveguides. The light intensity at the position of the neighbouring channels, i.e. 8 µm away from the channel, was one to two orders of magnitude lower for the SPIM-WGs. Further details are provided in Fig. 3b. The red curve, corresponding to conventional laser-written waveguides, clearly shows coupling into the neighbouring channels, evident from the multiple high light intensity peaks corresponding to the channel separation. In contrast, the SPIM-WGs exhibited minimal coupling to adjacent channels. We measured nearest-neighbour cross-talk of ≈3 × 10^–2^ for the conventional laser-written waveguides and ≈5 × 10^–4^ for the SPIM-WG channels. These measurements were repeated for multiple different chips, all of which showed consistent results, confirming the high reliability of the chip fabrication.

### Advanced mode matching and adiabatic mode conversion

**Fig. 4:**
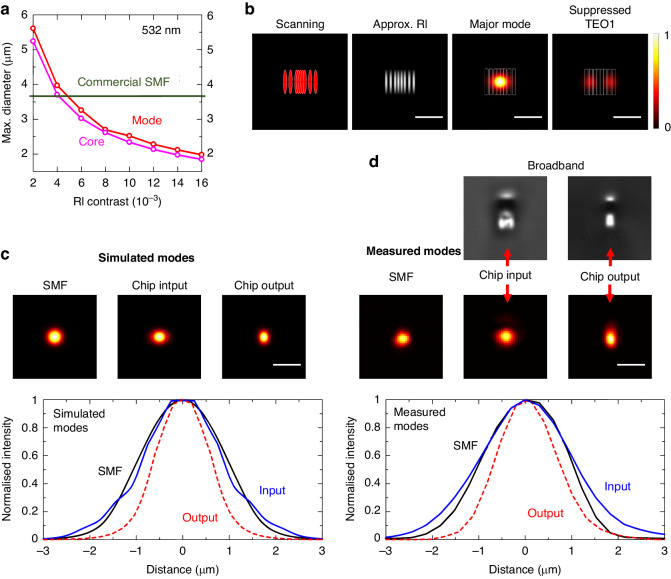
Enhancing chip coupling efficiency through advanced mode matching and adiabatic mode conversion. The size of the scale bars in (**b**–**d**) is 5 µm. **a** Calculated maximum waveguide core diameter to maintain single-mode guiding at 532 nm as a function of the RI contrast (core RI minus cladding RI). The maximum MFDs were determined from a COMSOL simulation. The cladding RI was 1.51 for borosilicate Eagle glass at 532 nm. Commercial SMFs typically have a cladding RI of 1.455 and a core RI of 1.4607. **b** Scanning scheme, approximate RI profile, and COMSOL-simulated guiding modes for a single SPIM-WG channel at the chip’s input facet. **c** Top: COMSOL-simulated laser guiding modes for an SMF, the output channel, and the input channel of the chip. Bottom: the dominant mode of the chip’s input channel compared to the SMF mode and the chip’s output mode. **d** Top: LED-illuminated microscope images of the chip’s output and input channels. Middle: experimentally measured 532 nm laser guiding modes for an SMF and for the SPIM channels. Bottom: intensity plots of the measured modes

To ensure that the required laser input power to the addressing setup scales favourably with the number of qubits, it is crucial to maintain a high throughput efficiency within the chip-based system. This has been a problem in previous single-ion addressing implementations, where losses of >10 dB have been observed due to poor coupling between system components^21^. The photonic chip must therefore implement high-efficiency coupling between the set of SMFs delivering the light, held in a VGA, to the input of the ion-trap lens system. This can only be achieved through effective mode matching between the components and adiabatic conversion between the modes. Figure 4a shows calculated maximum diameters for the waveguide core and guiding mode to maintain single-mode operation for different RI contrasts. Commercial SMFs typically have an MFD of 3.5–3.7 µm at a wavelength of 532 nm, while the SPIM-WG channel has a maximum single-mode MFD of 1.9 µm along the horizontal direction, owing to the high RI contrast of 0.015. Significant coupling losses would arise if the larger SMF modes are coupled directly to the smaller, high RI SPIM-WG channel modes.

An additional concern relates to position uncertainties in the VGA. The commercial VGAs we used were designed to have a core separation of 127 µm, but exhibited variable position offsets between the fibre cores of up to 0.7 µm along the *x*-direction (parallel to the linear fibre core array) and up to 0.3 µm along the y-direction (orthogonal to the linear fibre core array). These offsets significantly reduced the mode overlaps between the VGA and the photonic chip and increased the coupling losses. We developed a new design for advanced mode matching that effectively mitigates the impact of the VGA channel position variability. The design, presented in Fig. 4b, was built upon the central region of the four scans discussed earlier [Fig. 2b]. We introduced two additional scans on either side of the 4-scan SPIM-WG in order to increase the lateral size of the waveguide mode. The spacing of these additional scans was 1.5 times larger than that of the four central scans. As shown in Fig. 4b, this design considerably extended the lateral size of the dominant mode while effectively suppressing higher-order modes (e.g., the TE01 mode). Figure 4d includes broadband-light-illuminated microscope images of the channel input and output cross-sections, further highlighting the difference between RI modifications.

Figure 4c presents the simulated guiding modes for the SMF, the output channel (as described in the previous section), and the input channel (dominant mode only). The input channel mode was nearly identical to that of the SMF, but considerably larger than that of the output channel. The experimentally measured mode profiles, summarized in Fig. 4d, agreed well with the simulations. The measured mode for the input channel in Fig. 4d appeared slightly larger than the simulated dominant mode in Fig. 4c because the measured mode profile contained a superposition of multiple modes, while the simulation included only the dominant mode. Loss measurements indicated that this specialized design enhanced the coupling efficiency from <60% to ~80% and significantly improved the mode uniformity across the channels.

To address the disparity in cross-section between the channel input mode and the required output mode, we incorporated adiabatic mode conversion by changing the waveguide properties along the chip. Starting from the input facet, the RI profile was gradually changed over a total length of 2.2 mm, transitioning from the design illustrated in Fig. 4b to the design described in Fig. 2b. The RI profile from Fig. 2b was then maintained throughout the bending region until the chip output. A 3D render of the adiabatic mode conversion is presented in the “Materials and methods” section. The mode conversion efficiency was investigated through measurements of the losses of straight SPIM-WG channels with and without adiabatic mode conversion for >20 SPIM-WG channels. The losses were in the range 0.38 − 0.7 dBcm^-1^ (with typical 0.45 dBcm^-1^). No statistical significance was found in terms of the difference in propagation losses between the two designs, proving the practical effectiveness of adiabatic mode conversion using SPIM-WGs^25^.

### Integration with a trapped-ion quantum system

In our experiment we use trapped ^137^Ba^+^ ions as qubits. The ions are confined in a 3D monolithic microfabricated trap^30,31^ that allows for the generation of deep confining potentials while maintaining low heating rates, which is crucial for storing long ion crystals. The segmented electrode structure provides sufficient degrees of freedom for both ion shuttling across the trap and the generation of anharmonic potential shapes. The latter is particularly important for maintaining a uniform ion-ion spacing in large registers^32^, therefore increasing the minimum distance between neighbouring ions compared to harmonically spaced chains^8^. As explained in the previous sections, a larger ion-ion spacing reduces the cross-talk between neighbouring channels in the addressing setup.

The SPIM-WGs used for individual addressing in our setup were optimised for 532 nm. This wavelength enables driving Raman transitions within both the ground and the metastable level manifolds of the ^137^Ba^+^ ions (see the Supplementary information). The output of the photonic chip is mapped on the ion chain using a 2:1 lens relay, such that the distance between neighbouring beams at the ion position is reduced to 4 µm, matching our target ion spacing.

**Fig. 5:**
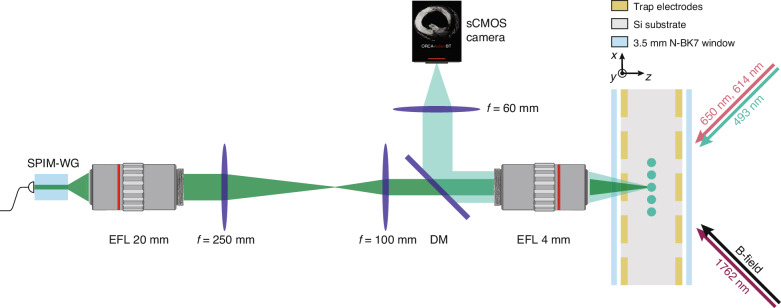
An overview of the trapped-ion setup. The ions are confined in a 3D monolithic microfabricated trap^30,31^. The 493 nm light scattered by the ions is collimated using a commercial NA0.5 microscope objective with an effective focal length (EFL) of 4 mm and refocused on an sCMOS camera for spatially resolved readout. The 532 nm light from the photonic chip is first collimated using a commercial microscope objective with an EFL of 20 mm. The focal lengths and positions of the subsequent *f* = 250 mm and *f* = 100 mm lenses are chosen to achieve the required 2:1 demagnification while also satisfying the geometrical constraints of the optical system. A dichroic mirror (DM) is used to overlap the 532 nm beam path with the 493 nm fluorescence beam path. This allows us to focus the 532 nm light on the ions with the same NA0.5 objective we use for imaging them. The applied magnetic field (*B*-field) is used to lift the degeneracy of the Zeeman states. The laser beams incident from the right are used for ion cooling, state preparation, and measurement, as well as for mapping to and from the metastable D_5/2_ level (see the Supplementary information for further details)

An outline of the optical system used to reimage the SPIM-WG output on the ions is shown in Fig. 5. The output of the chip is collimated using a high-quality commercial microscope objective to minimise aberrations. Another microscope objective is used to both refocus the 532 nm control light from the waveguide on the ions, and to collect the 493 nm photons scattered by the ions. The latter objective is chromatically corrected for visible wavelengths, and glass-thickness compensated for the glass window on the vacuum chamber. This allows us to both image and address the ions with minimal aberrations.

The VGA and SPIM-WG assembly is mounted on a stainless steel plate before integration with the rest of the optical system (see “Materials and methods”). This, combined with the fibre network used to interface between the laser source and the photonic chip as outlined in Fig. 1, enables simple exchange of chips in the ion trap system with minimal realignment.

### Cross-talk measurement using a single trapped ion

To characterise the performance of the complete individual addressing setup within the ion trap experiment, we measured the beams’ spatial profiles by using a single ion as a point-like sensor. When a single 532 nm beam is directed at the ion, it introduces an AC Stark shift on the quadrupole transition frequencies between states in the S_1/2_ and D_5/2_ levels. This shift is proportional to the intensity of the 532 nm light experienced by the ion. To characterise the spatial intensity distribution from the SPIM-WG output, we transported the ion from the trap centre to an axially displaced position *x* (see Fig. 5) and measured the quadrupole frequency shift as a function of *x*. We were thus able to measure the beam profiles, the beam spacing, and the cross-talk of the system.

For the purpose of this measurement we used the |S_1/2_, F = 2, m_F_ = 0〉↔ | D_5/2_, F = 4, m_F_ = +1〉 transition as it exhibits the lowest magnetic field sensitivity of all available transitions for the magnetic field direction and the beam geometry shown in Fig. 5. The frequency shift introduced by the 532 nm beam can be seen as a *Z*_*ϕ*_ rotation on the qubit state with the phase *ϕ* proportional to the magnitude of the AC Stark shift, and hence proportional to the beam intensity. To estimate this phase in a way that is robust to additive errors, such as those accumulated during state preparation and measurement, we used a robust phase estimation protocol^33,34^ (RPE). To enhance the system’s coherence time and hence increase the available probe duration (therefore also increasing the dynamic range of the measurement), we embedded the RPE sequence inside a Knill dynamical decoupling sequence^35^ (KDD). Further details on the sequences used can be found in the “Materials and methods” section.

**Fig. 6:**
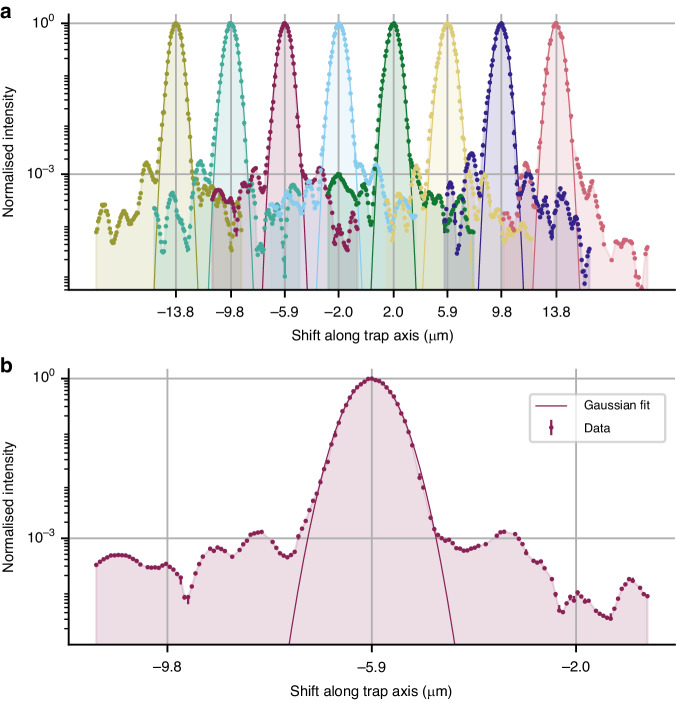
Intensity measurement of the 532 nm beams at the trap centre. Obtained by shuttling a single ion along the trap axis and measuring the AC Stark shift introduced on one of the quadrupole transitions between the S_1/2_ and D_5/2_ levels. The data from each of the channels is normalised relative to the maximum intensity of that channel. The lines are Gaussian fits to the data, giving a mean beam waist radius of 0.67±0.06 µm and a mean channel spacing of 3.95±0.03 µm. **a** A measurement of all 8 channels. The values on the *x*-axis correspond to the measured positions of the SPIM-WG channels relative to the trap centre. **b** A zoomed-in view of the third channel [left-to-right in (**a**)]. The values on the *x*-axis correspond to the measured positions of this channel and its nearest neighbours relative to the trap centre. In most cases the errorbars are smaller than the marker size

The results from this measurement are shown in Fig. 6. We measured a mean beam waist radius of 0.67 ± 0.06 µm and a mean channel spacing of 3.95 ± 0.03 µm. This is consistent with the 2:1 demagnification from our optical system (see Fig. 5). For all channels, the cross-talk level was below 10^–3^, with a lowest measured cross-talk of ∼ 10^–5^. The variation in cross-talk across the chip was most likely due to light leakage through the fibre AOMs as well as aberrations and/or scatter from the optical components in the beam path that affect the channels non-uniformly.

**Fig. 7:**
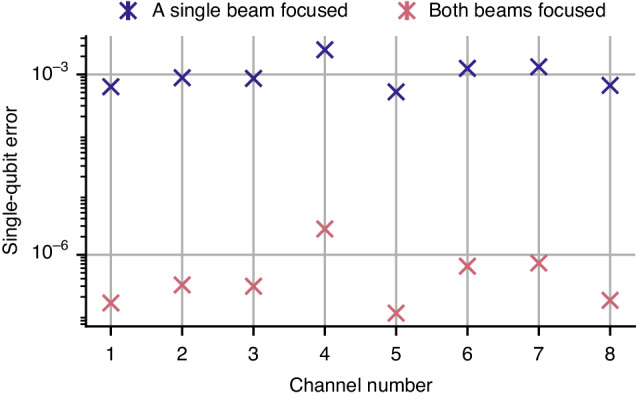
Errors introduced on the quantum state of the ‘idle’ ions in a computation due to intensity cross-talk within the individual addressing system. We plot the worst-case error for each ion position: we assume that we implement a π pulse on both ion *i-1* and ion *i* + *1*, and sum the two contributions to obtain the total error on ion *i* (except for the edge channels where there is a single contribution). We consider two possible configurations for driving a Raman transition: when only one of the two laser beams is focused on the individual ions and the second beam is global for the entire register; and when both Raman beams are focused on the individual ions. The errors and associated errorbars are calculated based on the measurements presented in Fig. 6. The errorbars are smaller than the marker size

In Fig. 7 we plot the error introduced to the state of the ‘idle’ ions during a computation for the data shown in Fig. 6. We consider two cases: when a single beam is focused down on the target ion, and the case of a Raman transition where both beams are focused on the target ion. The former is relevant when the qubit transition is itself an optical transition, or when a two-photon Raman process is used and one of the beams is global for the entire qubit register. For each channel we sum the nearest-neighbour contributions (except for the edge channels where there is a single contribution). In both cases this error is much lower than, or comparable to, state-of-the-art two-qubit gate errors^3,5,18^ and will therefore not limit the device performance. The cross-talk performance can be enhanced even further with well-known techniques such as coherent cancellation^36^ or composite pulse sequences^37^.

## Discussions

We have presented a scalable and configurable way for individual optical addressing in chains of trapped atomic ions. We have designed and fabricated an SPIM-WG chip that offers a very high RI contrast between the core and the cladding, therefore achieving very low cross-talk at a small channel separation. The photonic chip is coupled with low loss to a fibre network that enables individual phase, frequency, and amplitude control of each of the channels, thus enabling parallel operations. The photonic chip manufacturing procedure is very flexible in terms of the output pattern configuration, and can therefore be used to manufacture devices suited to most trapped-ion setups, including creating chips with a non-uniform channel spacing.

Measurements of the performance of our individual addressing system, optimised for 532 nm light, using a single trapped ^137^Ba+ ion showed cross-talk well below 10^–3^ across the chip. The corresponding estimated worst-case nearest-neighbour error is ∼10^–6^ if both beams are focused on a single ion or ∼10^–3^ if one beam is focused on the ion and the second beam is global for the register. In the latter case, well-known techniques such as composite pulses or coherent cancellation can be used to further reduce this error.

The procedure used to manufacture the SPIM-WGs is highly flexible and so it can be used to create devices with a large number of channels, as well as devices with non-uniform channel spacing. The latter capability is important for applications in ion traps that do not have sufficient degrees of freedom to generate anharmonic potentials to keep the ion spacing uniform across the ion chain, or where harmonic potentials are desired. Preliminary results from the characterisation of devices with 32 channels (see the Supplementary information) show no degradation of performance in terms of cross-talk and minimal change in propagation losses. In addition, the same technique can be used to manufacture devices optimised for use at other optical wavelengths and therefore be integrated into setups using different ion species and/or different gate mechanisms.

In conclusion, we have presented a method to individually optically address chains of trapped atomic ions, incorporating a fibre network coupled to a novel photonic chip. The photonic chip’s manufacturing procedure enables a very high refractive index contrast between the light guiding channels’ core and cladding, therefore maintaining a very low cross-talk between neighbouring channels. Our method achieves significantly lower cross-talk compared to existing individual optical addressing methods integrated with trapped-ion setups, while maintaining high scalability and flexibility.

## Materials and methods

### Photonic chip fabrication with adaptive optics ultrafast laser system

The optical system and 3D spatial coordinators are shown in Fig. S1. The ultrafast laser system used a regenerative amplified Yb:KGW laser (Light Conversion Pharos SP-06–1000-pp) with 1 MHz repetition rate, 514 nm wavelength and 170 fs pulse duration. A spatial light modulator (SLM, Hamamatsu Photonics X10468) was aligned and imaged by a 4-f lens system to the pupil of objective lens. The power of the laser beam was controlled with a motorized rotating half waveplate together with a polarizing beam splitter (PBS). The laser beam at the objective lens focus was circularly polarized. The glass sample was fixed on a three-axis air bearing stage (Aerotech ABL10100L/ABL10100L/ANT95–3-V) to control the movements.

The photonic chip was created in borosilicate glass (Corning EAGLE 2000). For all the results in this paper, if not otherwise specified, the chips were fabricated using a 0.5NA objective lens (93% transmission), at a depth of 120 µm from the surface of the glass sample, with scanning speed of 8 mms^-1^ and pulse energy (measured at the objective pupil) of 80 nJ.

For all fabrication runs in this paper, the spherical aberration arising from the refractive index mismatch between immersion and sample was corrected, but not included in the description. System aberrations arising from misalignments were calibrated by using a wavefront-sensorless method (cite), and were fully corrected by the SLM.

After laser fabrication, photonic chips were polished using a sequence of 30 µm, 9 µm, 3 µm and 1 µm polishing films. A layer of at least 150 µm of glass was polished off both the input and output facets of the chip.

### SPIM-WG chip integration into the ion trap setup

To couple light from the VGA to the photonic chip, we kept the chip fixed and placed the VGA on a 6-axis positioning stage as shown in Fig. S7. We imaged the chip output on a camera to evaluate the amount of light coupled from the VGA. We optimised the position of the VGA to maximise the average coupling efficiency, while simultaneously keeping the coupling efficiency across all channels as uniform as possible. Due to tolerances in the VGA spot size and spacing, the average coupling efficiency we achieved when optimising for all channels was lower than the maximum coupling efficiency that we could achieve for a single channel. In the devices used for the demonstration here, we achieved an average coupling efficiency of 45 ± 3%. While this coupling efficiency can be increased further by increasing the waveguide MFD (and hence enabling a larger overlap between the VGA mode and the waveguide mode in the presence of position errors), the waveguides used here already have the largest possible diameter to maintain single-mode operation at 532 nm (see Fig. 4 in the main text).

As outlined in the main text, the coupling between the VGA and the chip is extremely sensitive to relative position changes between the VGA and the chip. In addition to changes in the power in each channel, changes in the coupling efficiency can also modify the phase and/or polarisation of the light that lead to gate errors. To ensure the coupling between the VGA and the chip stays constant during system operation, after optimising the coupling between the two components, the VGA was glued to the chip using the NOA061 UV curing glue as shown in Fig. S7b. To avoid changes in the coupling while the glue was curing, we cured at a gradually increasing UV power over 2−3 h.

The VGA and photonic chip assembly was then glued to a stainless steel plate and was transported to the ion trap system. This stainless steel plate was then bolted on a positioning stage that is part of the setup as shown in Fig. S7c. Upon exchanging chips, the stainless steel plate with the glued VGA-photonic chip assembly is the only component that needs to be exchanged, while the remaining optical setup is left unchanged. Therefore only minimal realignment is necessary following chip exchange.

### Robust phase estimation for an AC Stark shift measurement

**Fig. 8:**
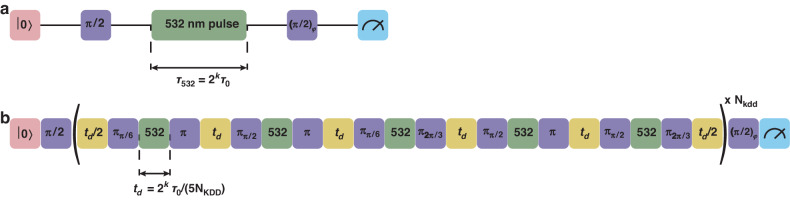
Pulse sequence for an AC Stark shift measurement. Unless otherwise specified, all π and π /2 pulses are rotations about the *x*-axis of the Bloch sphere. **a** Robust phase estimation (RPE) sequence for estimating the phase introduced on the |→〉 = ( | 0〉 + *i* | 1〉) /√2 state as a result of the AC Stark shift from a single 532 nm beam. The ion is prepared in the state |0〉 and the subsequent 1762 nm π/2 pulse prepares the state |→〉. Then a single 532 nm beam is turned on for a duration *τ*_532_ = 2*kτ*_0_ where *τ*_0_ is the base duration defining the maximum observable AC stark shift of δ_ac, max_ = 1/*τ*_0_. The final 1762 nm (π/2)_*ϕ*_ pulse defines the measurement basis. The measurement is performed for *ϕ* = 0, π/2, π, 3π/2 to minimise the effect of polarising noise in one direction. **b** The sequence used to measure the AC Stark shift introduced by a single 532 nm beam on the |0〉 ↔ | 1〉 transition, consisting of the RPE protocol embedded into a KDD sequence to extend the system coherence time

We used the AC Stark shift induced by a single 532 nm beam on the |S_1/2_, F = 2, m_F_ = 0〉 ↔ | D_5/2_, F = 4, m_F_ = +1〉 transition to measure the intensity of the beams and hence characterise the chip output at the ion position. This AC Stark shift introduces a relative phase between the two states that is proportional to the beam intensity. We used robust phase estimation^33,34^ (RPE) as shown in Fig. 8a to estimate that phase. This significantly reduced our sensitivity to other systematic errors such as state preparation and measurement errors. To reduce the sensitivity of the system to decoherence and enable longer probing durations for the RPE sequence (hence also increasing the measurement dynamic range) we embedded the RPE sequence as part of a Knill dynamical decoupling sequence^35^ (KDD) as shown in Fig. 8b. For probe durations shorter than the system coherence time, the RPE sequence was embedded in a spin-echo sequence instead. This avoided 532 nm pulses with durations comparable to the AOM settling time constants. For probe durations longer than the system coherence time, the KDD sequence in Fig. 8b was used and the number of KDD pulses NKDD was tuned to ensure that the maximum duration between consecutive π-pulses did not exceed the system coherence time. In this experiment, this duration was set to 500 µs.

### Supplementary information

Supplementary information
