# Supplementary material for: Low cross-talk optical addressing of trapped-ion qubits using a novel integrated photonic chip
Source: Light Sci Appl. 2024 Aug 20;13:199. doi: 10.1038/s41377-024-01542-x (PMC11335750; doi:10.1038/s41377-024-01542-x)
Supplement: Supplementary file 1 — Supplementary information [file 41377_2024_1542_MOESM1_ESM.pdf]

Supplementary information

Low Cross-Talk Optical Addressing of Trapped-Ion Qubits Using a Novel Integrated Photonic Chip

Ana S. Sotirova[1,+,*], Bangshan Sun[2,+,*], Jamie D. Leppard[1], Andong Wang[2], Mohan Wang[2], Andres Vazquez-Brennan[1], David P. Nadlinger[1], Simon Moser[3], Alexander Jesacher[3], Chao He[2], Fabian Pokorny[1], Martin J. Booth[2,*], and Christopher J. Ballance[1,*]

[1]University of Oxford, Department of Physics, Oxford, OX1 3PU, United Kingdom

[2]University of Oxford, Department of Engineering Science, Oxford, OX1 3PJ, United Kingdom

[3]Institute of Biomedical Physics, Medical University of Innsbruck, Müllerstraße 44, 6020 Innsbruck, Austria

*Corresponding to: Bangshan Sun (b.s.shawnsuen@gmail.com), or Ana S. Sotirova (ana.sotirova@physics.ox.ac.uk), or Martin J. Booth (martin.booth@eng.ox.ac.uk), or Christopher J. Balance (chris.ballance@physics.ox.ac.uk)

[+]These two authors contributed equally to this work.

Adaptive optics ultrafast laser system

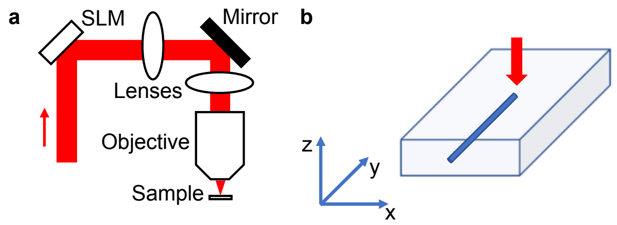

Figure S1 a A diagram of the ultrafast laser system for SPIM-WG fabrication. SLM: spatial light modulator. **b** A sketch to illustrate the 3D coordinates relative to the chip. The red arrow marks the laser propagation direction.

Microscopic imaging of the photonic chip

Supplementary Fig. S2 includes a system diagram for microscopic images and loss measurements. A lab-built LED-illuminated widefield transmission microscope (microscope 1) was used to check light channel cross sections after polishing. To image the laser guiding mode profiles, single mode fibres were used to guide laser light into the input facet of a given channel. The fibre output was mounted on and adjusted with a six-axis stage (three linear and three angular degrees of freedom). Another lab-built microscope (microscope 2) was used to monitor and measure the distance between the fibre tip and the chip input facet. During the measurement of the coupling losses, we brought the fibre tip very close (< 1 µm) to the photonic chip to avoid input beam expansion. The output facet of a channel was imaged by microscope 1 in order to capture the guided laser mode profile.

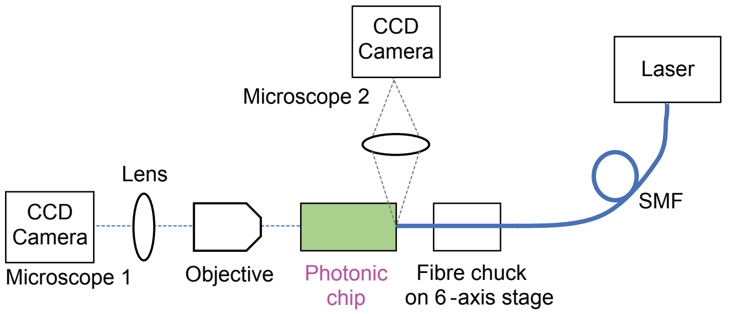

Figure S2 The system to image the photonic chip facets, obtain broadband microscopic images, laser mode profiles, and measure the photonic device losses. There were two lab-built microscopes: microscope 1 was used to obtain device facet images and mode profiles; microscope 2 was used for live monitoring and measurement of the distance between input fibre tip and chip facet.

Refractive index profile measurement

Refractive index profiles were measured with a lab-built 3D tomographic microscope[1]. The imaging system recorded many intensity images of the region of interest at different illumination angles ranging between about -45° and 45°. The light source was a collimated blue LED (460 nm). A two-dimensional refractive index cross-section of the waveguide was then reconstructed from the image stack using an error reduction algorithm based on gradient descent and simulated beam propagation.

Bending loss simulations

In Fig. S3 we plot the simulated bending losses as a function of the waveguide bending radius, calculated using an analytical model for step-index waveguides as described in Ref. 2 and 3. We used

our measured parameters of a refractive index contrast of 0.015 (refractive indices of the core and the cladding of 1.525 and 1.51, respectively), a core diameter of 1.8 µm, and a wavelength of 532 nm. Our design yielded curvatures between 31.2 mm and 217.8 mm for the eight waveguides. As can be seen in Fig. S3, the bending losses at such curvatures are negligible. At large bend radii, e.g. above 2 mm, as in our design, we expect that the losses are dominated by phenomena other than the bending loss. This agrees with our measurements, which did not show any measurable differences in losses between straight and curved SPIM-WG channels.

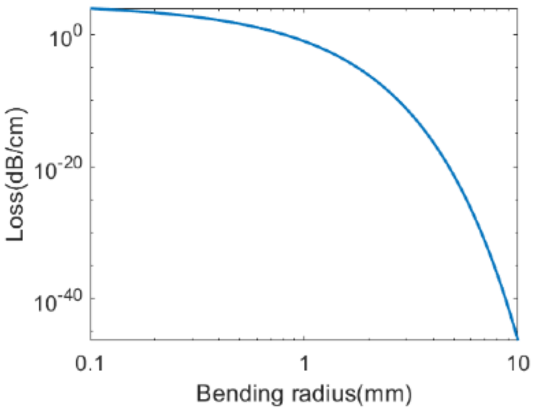

Figure S3 Simulation of the bending loss as a function of the bending radius.

Mode progression in the SPIM-WG chip

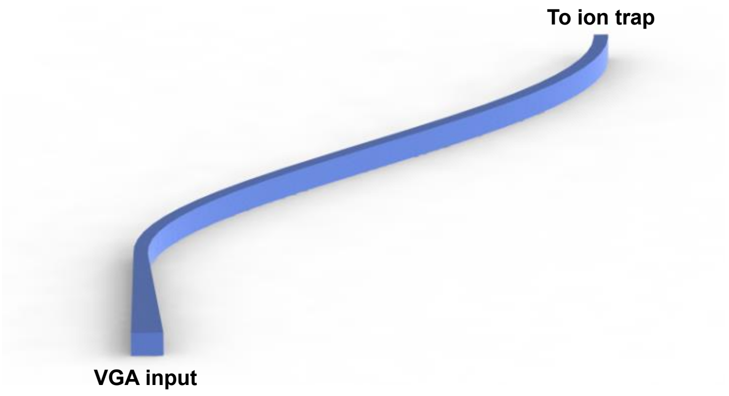

Figure S4 A 3D diagram of a single adiabatic mode converter channel in the photonic chip. The adiabatic mode conversion was implemented at the straight input region, while the channel cross-section at the bending region was kept constant until the channel output.

Preliminary results from 32-channel chips

We fabricated 32-channel chips using the same laser-written techniques and specifications as for the 8-channel chips presented in the main text of the paper (~8 µm channel spacing and ~2 µm MFD at the output). Microscopic images of such a chip are shown in Fig. S5.

The overall length of the 32-channel chip was double the length of the 8-channel chip, and the curved region was 17 mm. We measured propagation losses in the range 0.4–0.8 $dBcm^{-1}$ and no noticeable extra bending losses in the channels closer to the edge. Classical measurements of the chip performance indicated a cross-talk well below 10^{-3}, similar to the 8-channel chip presented in the main text.

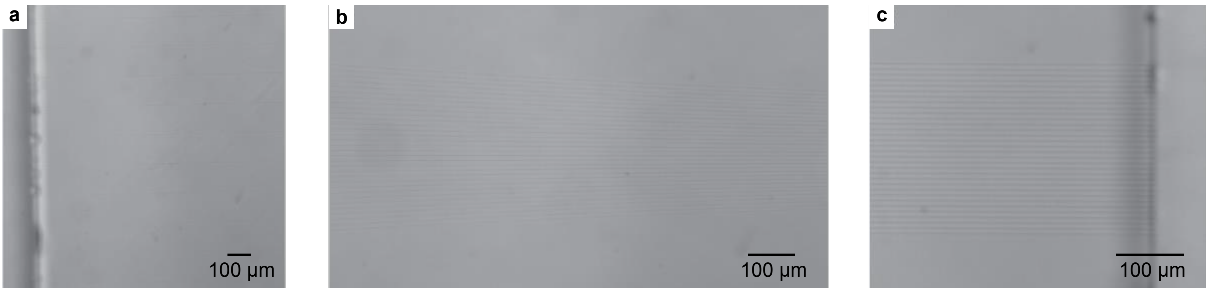

Figure S5 Top view microscopic images of a 32-channel photonic chip. The 32-channel chip was measured to have similar performance in terms of cross-coupling and losses to the 8-channel chip described in main text. **a** Image at the chip input, where the channel are spaced by 127 µm to match the VGA. **b** Image at the middle part of the chip where the channel spacing is reduced. **c** Image at the chip output, where the channel spacing is reduced to 8 µm.

Barium level structure

The chips we manufactured are optimised to work with barium ions: we can use 532 nm light to drive the qubit transition for both ground $S_{1/2}$ level and metastable $D_{5/2}$ level qubit encodings as shown in Fig. S6a. An overview of the barium level structure is shown in Fig. S6b. The 493 nm cycling transition is used to cool the ions and readout their state. The 650 nm laser is used to pump population out of the $D_{3/2}$ level. We use a 1762 nm laser to map between the ground and the metastable level qubits. We also use one of the available 1762 nm transitions to measure the light shift, and hence the intensity, from the beams generated by our individual addressing setup (see the main text). The 614 nm laser is used to deshelve population from the metastable $D_{5/2}$ level at the end of an experiment sequence.

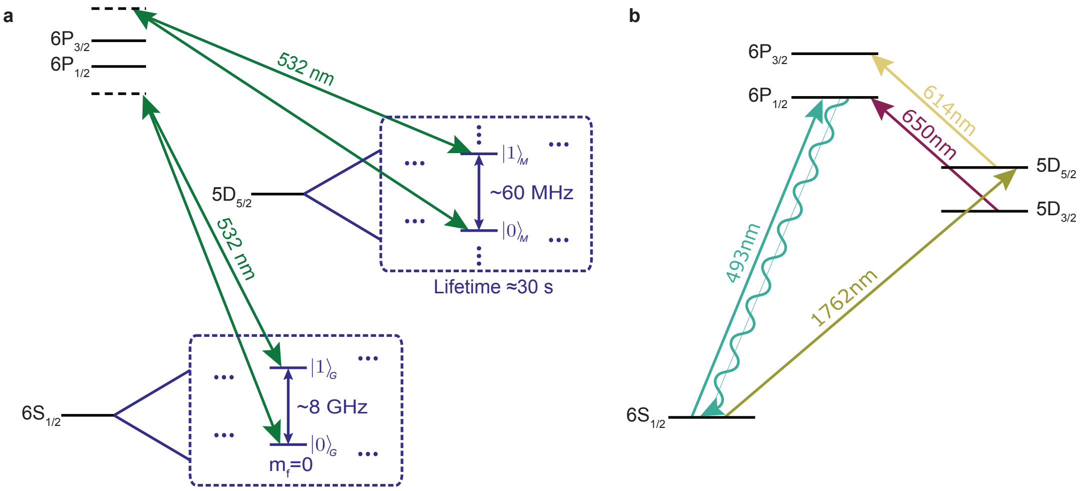

Figure S6 a Level structure of $^{137}Ba^{+}$ showing the ground $S_{1/2}$ and metastable $D_{5/2}$ level qubit encodings. Both types of qubits can be driven with a two-photon Raman process using 532 nm light. The lifetime of the metastable level is 30.14 s[4], much longer than the typical timescale of the operations, making it a suitable place to encode qubits in addition to the ground level. **b** A high-level overview of the barium level structure. The ions are cooled and detected by repeatedly exciting the transition between the $S_{1/2}$ and the $P_{1/2}$ levels and collecting the scattered 493 nm light. The 650 nm laser is used to repump population from the $D_{3/2}$ level. The 1762 nm laser is used to map population to and from the metastable $D_{5/2}$ level and convert between the two qubit types. The 614 nm laser deshelves population from the $D_{5/2}$ level at the end of the experiment.

Coupling between the VGA and the photonic chip

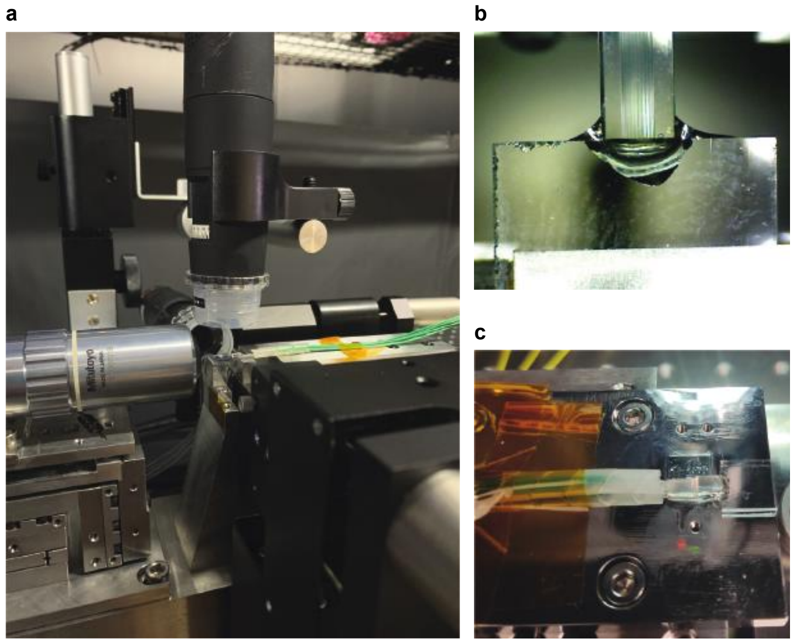

Figure S7 Coupling between the VGA and the photonic chip. **a** Setup used to couple light from the VGA into the photonic chip. The VGA was placed on a 6-axis positioning stage (right) while the photonic chip was held stationary (centre). The objective on the left was used to image the photonic chip output on a camera to evaluate the quality of the coupling. **b** A photo of the VGA glued to the photonic chip after the coupling was optimised. **c** A photo of the VGA and photonic chip assembly glued on a stainless steel plate prior to integration with the rest of the optical system in the ion trap setup.

References

1. Barr., N. et al. Tomographic refractive index profiling of direct laser written waveguides. Optics Express 29, 35414–35425, DOI: 10.1364/OE.434846 (2021).
2. Marcuse, D. Curvature loss formula for optical fibers. Journal of the Optical Society of America 66, 216–220, DOI: 10.1364/JOSA.66.000216 (1976).
3. Snyder, A. W. & Love, J. D. Optical Waveguide Theory (Boston, MA, 1984).
4. Zhang, Z. et al. Branching fractions for $P_{3/2}$ decays in Ba^{+}. Physical Review A 101, 062515 (2020).
